# Genomic and metabolic features of *Bacillus cereus*, inhibiting the growth of *Sclerotinia sclerotiorum* by synthesizing secondary metabolites

**DOI:** 10.1007/s00203-022-03351-5

**Published:** 2022-12-01

**Authors:** Jinghan Hu, Baozhu Dong, Dong Wang, Huanwen Meng, Xiaojuan Li, Hongyou Zhou

**Affiliations:** 1 College of Horticulture and Plant Protection, Inner Mongolia Agricultural University, Hohhot, 010020 Inner Mongolia China; 2 Inner Mongolia Cold and Arid Region Crop Protection Engineering Technology Center, Hohhot, 010020 Inner Mongolia China

**Keywords:** *Bacillus cereus*, Genome mining, Secondary metabolites, *Sclerotinia sclerotiorum*, Antifungal activity

## Abstract

We investigated the biocontrol mechanism of *Bacillus cereus* CF4-51 to find powerful microbes that effectively control *Sclerotinia sclerotiorum.* To assess its inhibitory effect on fungal growth, the plant pathogen (*S. sclerotiorum*) was co-cultured with *Bacillus cereus*. Scanning electron microscope (SEM) was used to study the morphology of *S. sclerotiorum* treated with CF4-51 biofumigant. The expression of sclerotium formation-related genes was analyzed by qRT-PCR. We performed whole genome sequencing of CF4-51 by PacBio Sequel platform. Lipopeptides were extracted from strain CF4-51 according to the method of hydrochloric acid precipitation and methanol dissolution. The volatiles CF4-51 were identified using gas chromatography–mass spectrometry (GC–MS). We found that the volatile organic compounds (VOCs) released by CF4-51 damaged the *S. sclerotiorum* hyphae and inhibited the formation of sclerotia. The qRT-PCR data revealed the down-regulated expression of the genes involved in sclerotial formation. Moreover, we analyzed the *B. cereus* CF4-51 genome and metabolites. The genome consisted of 5.35 Mb, with a GC content of 35.74%. An examination of the genome revealed the presence of several gene clusters for the biosynthesis of antibiotics, siderophores, and various other bioactive compounds, including those belonging to the NRPS-like, LAP, RIPP-like, NRPS, betalactone, CDPS, terpene, ladderane, ranthipeptide, and lanthipeptide (class II) categories. A gas chromatography–tandem mass spectrometry analysis identified 45 VOCs produced by strain CF4-51. Among these, technical grade formulations of five were chosen for further study: 2-Pentadecanone, 6,10,14-trimethyl-,1,2-Benzenedicarboxylic acid, bis(2-methylpropyl) ester, Dibutyl phthalate, Cyclododecane, Heptadecane. the five major constituents play important roles in the antifungal activity of the VOCs CF4-51 on the growth of *S. sclerotiorum*. The secondary metabolites produced by strain CF4-51are critical for the inhibition of *S. sclerotiorum* hyphal growth and sclerotial formation.

**Supplementary Information:**

The online version contains supplementary material available at 10.1007/s00203-022-03351-5.

## Introduction

*Sclerotinia sclerotiorum* is a worldwide plant pathogen that causes white rot on many economically important crops, including oilseed rape, lettuce, carrot, sunflower, pea, and beans, resulting in substantial losses annually (Bolton et al. 2010). Sclerotinia rot of many crops are controlled by chemical treatments. However, over-reliance on chemical pesticides to manage plant diseases can lead to several negative consequences, including the development of fungicide-resistant pathogens, the accumulation of pesticide residues in the environment, and the resurgence of pests and pathogens (Ongena and Jacques 2008). As a result, there is growing interest in biocontrol of plant diseases because it is more environmentally friendly. Plant growth-promoting rhizobacteria (PGPR) are potential biocontrol resources, including many soil bacteria that colonize the rhizosphere. A previous study revealed that PGPR may be useful for increasing the seed germination rate, plant biomass, and crop yield as well as for controlling plant diseases (Ping and Boland 2004). The application of PGPR is one of the most important ways of decreasing the use of chemical pesticides in sustainable agriculture development (Lucy et al. 2004).

*Bacillus* spp. are important PGPR that are widely used to regulate microorganism diversity, inhibit soil-borne plant diseases, and promote plant growth (Araújo et al. 2005). Moreover, most *Bacillus* spp. are potential biocontrol resources that are highly adaptable to environmental conditions (Romero et al. 2007; Huang et al. 2010). Earlier research demonstrated that many *Bacillus* strains can colonize all plant organs and protect plants against pathogens (Emmert and Jo 2010). There are several reports describing the use of *Bacillus cereus* and its metabolites as biological control materials that can effectively prevent diseases adversely affecting crop production (Silo-Suh et al. 1994; Stabb et al. 1994; Osburn et al. 1995; Lalloo et al. 2010; Xu et al. 2014). *Bacillus cereus* strain BS14, which was isolated from the *V. mungo* rhizosphere in district Saharanpur, Uttar Pradesh, India, is reported to have strong inhibitory effects for biocontrol of *M. phaseolina* (Kumar et al. 2021). Antibiotic metabolites were subsequently extracted from many *Bacillus* strains, the most important of which were the antifungal volatiles capable of degrading structural polymers (e.g., chitinase and β-1,3-glucanase) and inhibiting hyphal growth. Many antifungal volatiles have been identified, including 2-ethyl-hexanol, 2,4-bis(2-methylpropyl)-phenol, 4-hydroxybenzaldehyde, and 2-nonanone (Almenar et al. 2007). To date, 14 different volatile organic compounds (VOCs) produced by *Bacillus subtilis* have been identified. The VOCs with antifungal effects include 2,3,6-trimethylphenol, nonan-2-one, decan-2-one, dodecan-2-one, undecan-2-one, and 2-methylpyrazine. The effective protection against fungi provided by VOCs often involves the combined activities of many compounds. *Bacillus subtilis* produces non-volatile or volatile substances that have inhibitory effects against *Fusarium oxysporum* (Minerdi et al. 2009; Yuan et al. 2012), *Botryosphaeria berengeriana* (Zhang et al. 2010), *Trichoderma* sp. (Zheng et al. 2013), *Colletotrichum gloeosporioides* (Lee et al. 2012; Sungpueak et al. 2013), and *Penicillium* sp. (Andersen et al. 1994).

Many antifungal metabolites of PGPR can be exploited for controlling plant diseases. The application of PGPR can enhance plant growth because of the associated protection against various phytopathogens (Olanrewaju et al. 2019). Thus, biological control methods involving PGPR have emerged as alternatives to traditional management strategies for minimizing the use of conventional agricultural inputs to increase crop yield and quality. Previous study has demonstrated that PGPR are widespread in the rhizosphere, where they have crucial functions related to the control of phytopathogens (Liu et al. 2017). Genome analysis revealed many gene clusters involved in the synthesis of antifungal agents (Kamada et al. 2014). Draft genome sequence of *B. cereus* strain CITVM-11.1, including 5752 predicted protein-coding sequences some of which were involved in plant–bacteria interactions and contribute to the strong antagonistic activity against the charcoal root rot pathogen, *Macrophomina phaseolina* (Caballero et al. 2018). *Bacillus cereus* E41 genome contains a complete gene cluster for the lantibiotic thusin (thsA1TM1A2A2 = M2FE), which is predicted to produce the siderophore petrobactin (asbABCDEF) (Daas et al. 2017). *Bacillus velezensis* AK‑0 genome includes eight potential gene clusters associated with the biosynthesis of secondary metabolites (Kim et al. 2021). Genomes can be accurately assembled using long PacBio reads before being sequenced (Koren et al. 2013; Hutchison et al. 2016). The application of PacBio technology has been important for microbial genome studies and for identifying the genes contributing to the biocontrol-related functions of PGPR (Land et al. 2015). A previous comparative genome analysis detected many *B. subtilis* XF-1 gene clusters involved in the synthesis of antifungal metabolites (e.g., lipopeptides, polyketides, siderophores). A subsequent study indicated that there are two pathways for the synthesis of volatile growth-promoting compounds (Guo et al. 2015). A recent study investigated the utility of *Bacillus halotolerans* KLBC XJ-5 for the biocontrol of gray mold of harvested strawberry caused by *B. cinerea* as well as the underlying mechanism. Genome sequencing and bioinformatic analyses suggested that strain KLBC XJ-5 includes six antimicrobial BGCs in addition to four glycoside hydrolase family 18 gene clusters involved in chitin degradation (Wang et al. 2021).

Previously, we isolated a *B. cereus* strain CF4-51 from the rhizosphere of sunflower plants. The volatiles produced by *B. cereus* CF4-51 can significantly inhibit the formation of sclerotia of *S. sclerotiorum*. In this study, the effects of strain CF4-51 on the hyphal surface structure of *S. sclerotiorum* was investigated and the expression of genes related to sclerotia formation was analyzed. Furthermore, the genome and metabolites of *B. cereus* CF4-51. were investigated: (1) to identify the antifungal activity of *Bacillus cereus* CF4-51; (2) to identify the VOCs produced by *B. cereus* CF4-51; (3) to determine whether volatiles influence the expression of sclerotium formation-related genes.

## Materials and methods

### Strains and culture conditions

*Bacillus cereus* CF4-51 was previously isolated from the sunflower rhizosphere in Inner Mongolia Autonomous Region, China. Its identity was determined on the basis of morphological, physiological, and 16S rDNA sequencing data (GenBank accession No. CP063158–CP063161). The strain was maintained in LB medium supplemented with 30% glycerol and was kept at − 80 °C for long-term storage. The strain was subcultured on fresh LB agar slants at 30 °C for up to 24 h prior to use. The *S. sclerotiorum* strain used in this study was cultured on PDA at 20 °C and stored on PDA slants at − 80 °C.

### Strain CF4-51 whole-genome sequencing and assembly

Strain CF4-51 was cultured in LB medium at 30 °C and 150 rpm. When the bacteria reached the late exponential phase (about 8 h), cells were collected by centrifugation at 8000 rpm for 10 min and then washed twice with 0.9% NaCl solution. Genomic DNA was extracted from the cells using the Bacterial DNA Kit (Tiangen, Beijing, China). The quality of the extracted DNA was assessed by agarose gel electrophoresis, whereas the DNA concentration was determined using the Qubit fluorometer (Thermo Fisher Scientific, USA). The genome was sequenced using the PacBio RS II DNA Sequencing System (Pacific Biosciences, Menlo Park, CA, USA). The chromosome and plasmids were assembled using the software package of SMRT portal version 3.2.0 (260 × coverage).

### Genome annotations and features

Gene annotation were predicted using the self-training program GeneMark (Disz et al. 2010). Transfer RNA genes were predicted using the tRNAscan-SE version 1.3.1 (Lowe and Eddy 1997). Ribosomal RNA genes were analyzed using RNAmmer version 1.2 (Karin et al. 2007). Small nuclear RNAs were predicted by screening the Rfam database using the BLAST algorithm and then verified using cmsearch version 1.1rc4 (Burge et al. 2013). The CRISPRdigger program version 1.0 was used to detect CRISPR elements (Ge et al. 2016). The proteins encoded by the predicted genes were classified and the clusters of orthologous groups (COGs) were analyzed using the Pfam (Finn et al. 2016), Gene Ontology (GO) (Ashburner et al. 2000), and COG (Galperin et al. 2015) databases. The metabolic pathways of strain CF4-51 were analyzed using the KEGG database (Gerlich and Neumann 2000). Secondary metabolite biosynthetic gene clusters in strain CF4-51 were detected using antiSMASH (http://antismash.secondarymetabolites.org) (Kai et al. 2013; Medema et al. 2011).

### Inhibitory effects of *B. cereus* on *S. sclerotiorum* mycelial growth

To test the ability of *B. cereus* to inhibit *S. sclerotiorum* mycelial growth, a mycelial agar plug (5 mm diameter) containing actively growing hyphae was transferred from a 1-day-old PDA culture to the center of a plate (9 cm diameter) containing 10 mL PDA. Plates were incubated at 20 °C for 3 days. The experiment was repeated three times. To assess its inhibitory effect on fungal growth, CF4-51 was cultured in LB medium for 36 h at 30 °C and 170 rpm. The bacterial culture was centrifuged (8000 rpm for 10 min) and the cell-free culture was considered the supernatant. The supernatant was filtered (0.22 μm pores) and then added to PDA medium at a final concentration of 5% and 10%. For the control, LB liquid medium was added to the PDA medium. The growth rate of *S. sclerotiorum* was calculated after a 2-day incubation at 20 °C. Fungal growth inhibition (%) was calculated using the following formula: (*R*_c_ − *R*_t_)/*R*_c_ × 100; where *R*_c_ is the diameter of the control colony and *R*_t_ is the diameter of the colony treated with the supernatant.

### Antagonistic activity of *B. cereus* CF4-51 VOCs against *S. sclerotiorum*

A fungal plug (5 mm diameter) was placed on PDA medium in a bipartite Petri dish. The LB medium on the other side was coated with a CF4-51 bacterial solution. The Petri dish was double sealed with Parafilm and incubated at 28 °C for 3 days. The inhibition of mycelial growth (%) was calculated using the equation provided above. LB liquid medium was used as the control. Each experiment comprised three replicates and the experiments were performed in triplicate.

### Extraction and identification of the strain CF4-51 lipopeptide antimicrobial substances

Lipopeptides were extracted from strain CF4-51 according to a published method involving hydrochloric acid precipitation and methanol dissolution (Wei et al. 2010). Strain CF4-51 was cultured in 5 mL LB medium at 30 °C and 180 rpm and then 1% of the culture was used to inoculate 200 mL Landy medium (5 g/L l-Glutamic acid, 20 g/L glucose, 1.02 g/L MgSO_4·_7H_2_O, 1 g/L KH_2_PO_4_, 0.5 g/L KCl, 0.15 mg/L FeSO_4·_7H_2_O, 5 mg/L MnSO_4_, 0.16 mg/L CuSO_4_·5H_2_O, FeSO_4_ 1 mM, CuSO_4_ 6.4 mM, MnSO_4_ 0.29 M), which was incubated for 48 h at 30 °C and 180 rpm. The culture was centrifuged (8000 rpm for 20 min at 4 °C). The supernatant was collected and the pH was adjusted to 2.0–2.5 using 5 M HCl. After overnight incubation at 4 °C, the precipitates were collected by centrifugation at 8000 rpm for 20 min. The precipitates were dried on an ultra-clean workbench and then treated twice with 50 mL methanol (i.e., extraction solution). These extractions were combined to obtain the lipopeptide crude extract. We weighed the lipopeptide extract after drying, and prepared 10 mg/L lipopeptide extract with methanol. The antagonistic effects of the lipopeptide crude extract on *S. sclerotiorum* were analyzed according to the Oxford cup method (Fang et al. 2018). Briefly, 1% PDA medium (1 L medium containing 10 g agar) was added to the lower plate and allowed to solidify. Next, 100 mL 0.8% PDA medium (11medium containing 8 g agar) was added to the solidified PDA and then an *S. sclerotiorum* agar plug with 5 mm in diameter was placed at the center of the PDA plate. After the medium solidified, two sterile Oxford cups were placed on the prepared double-layered medium. The lipopeptide crude extract (150 μL) was added to each Oxford cup. The Oxford cup with 150 μL methanol in it was used as the control, Each experiment comprised three replicates and the experiments were performed in triplicate. After incubating at 25 °C for 5–7 days, the antagonistic effects of the lipopeptide crude extract were examined.

The strain CF4-51 lipopeptide crude extract was filtered (0.22 μm pores). The AKTA Purifier 10 fast protein liquid chromatography (FPLC) system was used to separate and purify the anti-microbial compounds in the filtrate. The FPLC system included the Source 5RPC St 4.6/150 column. Mobile phase A was an aqueous solution comprising 0.065% trifluoroacetic acid and 2% acetonitrile, whereas mobile phase B consisted of 0.05% trifluoroacetic acid and 80% acetonitrile. The detection wavelength was 215 nm and the flow rate was 1 mL/min. The gradient elution was performed as follows: 100% to 0% mobile phase A within 50 min. The anti-microbial compounds in strain CF4-51 were identified on the basis of the reported molecular weights of *B. subtilis* antibiotic lipopeptides (Arguelles-Arias et al. 2009).

### Extraction and identification of *B. cereus* CF4-51 VOCs

To analyze the *B. cereus* VOCs, 15 mL LB medium in a 100 mL flask was inoculated with strain CF4-51. After a 4-day incubation at 37 °C, the samples were collected and analyzed. The LB medium without inoculation of bacterium was used as the control. The VOCs were analyzed via solid phase microextraction (SPME) and gas chromatography–tandem mass spectrometry (GC–MS/MS). The SPME fiber (65 µm divinylbenzene/carboxen/polydimethylsiloxane fiber) was inserted into the headspace of the flask, which was incubated at 37 °C for 7 h. Compounds were then desorbed for 20 min in the injection port of the gas chromatograph at 220 °C with the purge valve off (splitless mode). An HP-5 capillary column (30.0 m × 0.25 mm × 0.25 µm, Thermo) and helium as the carrier gas were used for the GC–MS/MS. A Thermo Trace 1300 ISQ MS system was used for separating and detecting peaks. Each run was 45 min long. The initial oven temperature (40 °C) was held for 4 min. The temperature was then increased at a rate of 5 °C/min to 150 °C. After holding for 1 min, the temperature was increased at a rate of 10 °C/min to 280 °C and held for 5 min. The mass spectrometer was operated in the electron ionization mode at 70 eV with a source temperature of 280 °C for a continuous scan (35–400 m/z). The analysis was performed in the full-scan mode. Mass spectral data of the VOCs were compared with the data in the National Institute of Standards and Technology Mass Spectrum Library.

### Antagonistic activity of screened VOCs against *S. sclerotiorum*

The five major pure components of the VOCs (2-Pentadecanone, 6,10,14-trimethyl-,1,2-Benzenedicarboxylic acid, bis (2-methylpropyl) ester, Dibutyl phthalate, Cyclododecane and Heptadecane) (Macklin Biochemical Technology Co., Ltd, Shanghai, China) were selected to be verified potential mycelial inhibition. Mycelial growth inhibition assay was performed with bipartite Petri dish. A 5-mm-diameter fungal plug was placed on the PDA compartments, and a 5-mm-diameter sterilized filter paper absorbing the VOCs solution with concentration of 1, 10, 100, and 1000 μL/L were placed on the other side. Then, the bipartite Petri dish was sealed with parafilm and incubated at 20 °C, darkness for 3 days. The percentage inhibition of mycelial growth was calculated according to the equation described above. Filter papers with equivalent volume of sterile distilled water were used as control. Each experiment consisted of three replicates and the experiments were repeated twice.

### Effects of strain CF4-51 VOCs on *S. sclerotiorum* mycelial cells

The morphologies of the control *S. sclerotiorum* mycelia or the mycelia treated with CF4-51 VOCs were examined by scanning electron microscopy. To observe fungal structural changes, *S. sclerotiorum* was co-cultured with strain CF4-51 VOCs in a Petri dish sealed with tape for 6 days at 20 °C. The mycelia were harvested and fixed in 2% glutaraldehyde at 4 °C and then dehydrated using a series of ethanol solutions (30%, 50%, 80%, 90%, and 100%). The ethanol was replaced by 100% tertiary butyl alcohol. The cells were then lyophilized, coated with gold, and examined using the S-3500 N field emission scanning electron microscope (Hitachi, Tokyo, Japan).

### Quantitative real-time polymerase chain reaction (qRT-PCR)

The expression of sclerotium formation-related genes was analyzed by qRT-PCR. Total RNA was extracted from *S. sclerotiorum* cells co-cultured with strain CF4-51 VOCs for 5 days using the TransZol Up Plus RNA Kit (TransGen Biotech, Beijing, China). First-strand cDNA was obtained using reverse transcriptase (TransGen Biotech) and random hexamer primers. The qRT-PCR analysis was performed using SYBR Premix Ex Taq (TransGen Biotech). Each 20-μL reaction volume included 10 μL SYBR Premix Ex Taq, 8 μL nuclease-free water, 0.5 μL 10 mM primers (forward and reverse), and 100 ng cDNA. The PCR program was as follows: 95 °C for 30 s; 40 cycles of 95 °C for 5 s and 60 °C for 30 s. Relative gene expression levels were calculated according to the 2^−*ΔΔCt*^ method (Lv et al. 2015). The β-tubulin gene was used as an internal control. Details regarding the qRT-PCR primers are listed in Supplementary Table S1.

### Statistical analysis

Data were analyzed using Excel 2013 (Microsoft Corporation, Redmond, WA, USA) and SPSS software (version 23.0; IBM Corp., Armonk, NY, USA). The significance of any differences among treatments was determined using Duncan’s new multiple range test.

## Results

### Features and genome annotations of the *B. cereus* strain CF4-51

**Fig. 1:**
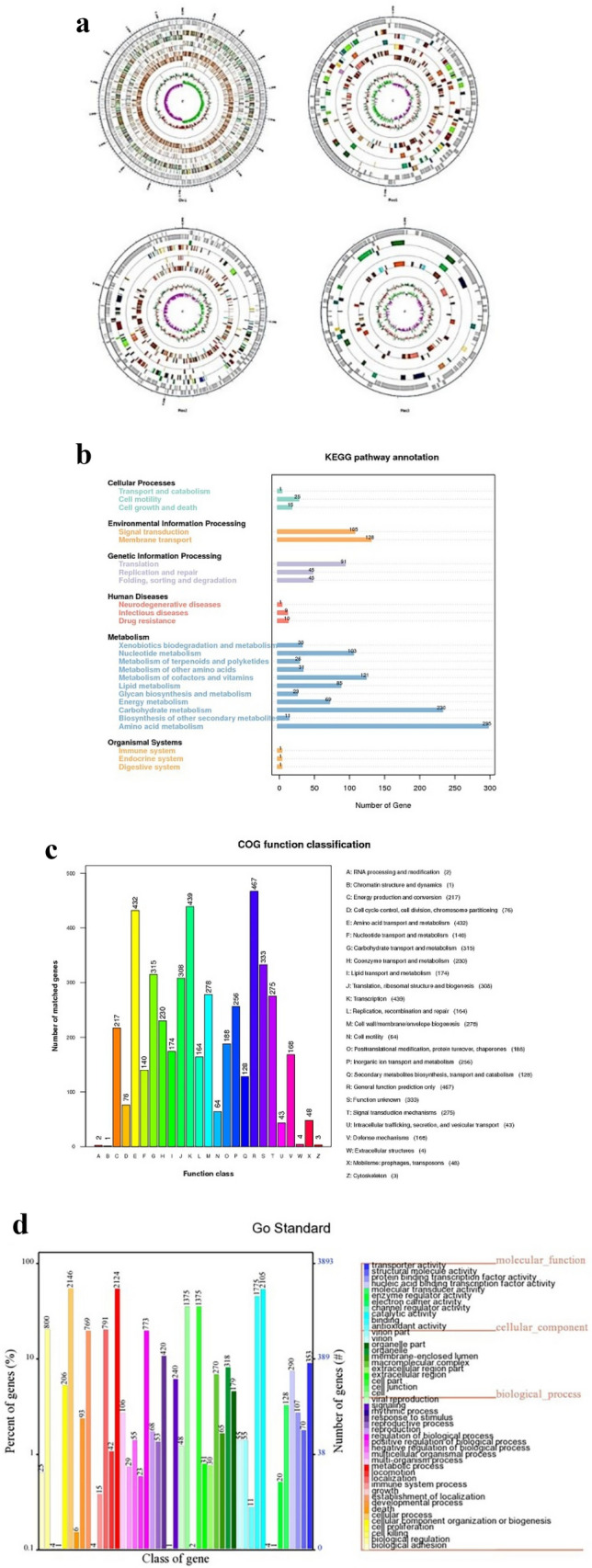
Genome annotations and features. **a** Details regarding the *Bacillus cereus* CF4-51 genome. **b** Functional annotation of *B. cereus* CF4-51 genes according to the KEGG database. **c** Functional annotation of *B. cereus* CF4-51 genes according to the COG database. **d** Functional annotation of *B. cereus* CF4-51 genes according to the GO database.

We analyzed the CF4-51 genome to screen for gene clusters involved in anti-microbial metabolites synthesis. The CF4-51 genome comprised 5.35 Mb, with a GC content of 35.74%. The three plasmids included in the genome consisted of 0.19, 0.38, and 0.08 Mb. An analysis using GeneMark tools resulted in the detection of 6166 predicted genes (Fig. 1a).

Of the genes in the *B. cereus* CF4-51 genome, 4144 were classified according to the COG database. The gene annotation results indicated that 128 genes are involved in the biosynthesis, transport, and catabolism of secondary metabolites. Moreover, 431, 439, and 217 genes contribute to amino acid transport and metabolism, enzyme transport and metabolism, and energy production and conversion, respectively. In addition, 315 genes influence carbohydrate transport and metabolism. Furthermore, 467 genes were assigned to the general function prediction category, whereas 333 genes had unknown functions (Fig. 1c).

A total of 3892 *B. cereus* CF4-51 genes were annotated with GO terms (Fig. 1d). To further characterize the gene functions, a KEGG pathway enrichment analysis was performed, during which 2598 genes were assigned to 151 metabolic pathways. More specifically, 41, 233, 181, and 20 genes are involved in cellular processes, environmental information processing, genetic information processing, and human diseases, respectively. Regarding the enriched metabolic pathways, we identified 45 genes related to DNA replication and repair, which affect bacterial viability. Another 26 genes are related to terpenoid and polyketone metabolism. Hence, these genes may participate in the synthesis of anti-microbial metabolites (Fig. 1b).

### Analysis of gene clusters involved in the synthesis of secondary metabolites

**Fig. 2:**
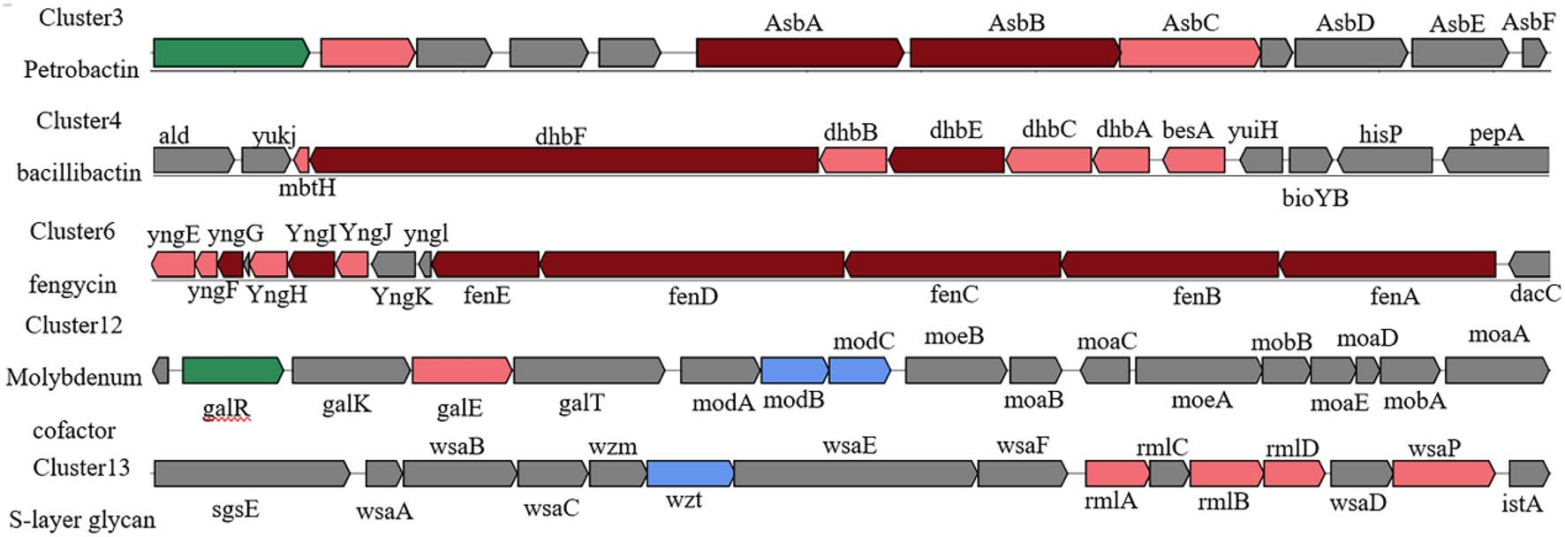
Schematic diagram of five secondary metabolite biosynthetic gene clusters in *B. cereus* CF4-51

The gene clusters involved in secondary metabolite synthesis predicted using antiSMASH (Table 1) included the following: NRPS-like, LAP, RIPP-like, siderophore, NRPS, betalactone, CDPS, terpene, ladderane, ranthipeptide, and lanthipeptide (class II). The structures of the gene clusters were partially characterized (Fig. 2). These clusters comprised core biosynthetic, additional biosynthetic, transport-related, regulatory, and other genes. Cluster 3 was homologous with the cluster involved in petrobactin synthesis. Cluster 4 was similar to the bacillibactin synthetase cluster, cluster 6 was similar to a fengycin biosynthetic cluster in *B. velezensis* strain FZB42, cluster 11 was similar to a polyoxypeptin synthetase gene cluster, cluster 12 was similar to a molybdenum cofactor synthetase cluster, and cluster 13 was similar to an S-layer glycan synthetase cluster. These findings indicate that strain CF4-51 produces bacteriostatic compounds.

**Table 1:** Secondary metabolite-related gene clusters in *Bacillus cereus* CF4-51 identified using antiSMASH

Region	Type	From	To	Most similar known cluster	Similarity (%)	MIBiG BGC-ID^a^
Region 1.1	NRPS-like	410,445	453,839	–	–	
Region 1.2	LAP, RIPP-like	1,211,941	1,235,447	–	–	
Region 1.3	siderophore	1,883,046	1,896,753	Petrobactin	100	BGC0000942
Region 1.4	NRPS	2,167,093	2,216,034	Bacillibactin	46	BGC0000309
Region 1.5	NRPS	2,337,375	2,399,951	–	–	
Region 1.6	betalactone	2,476,367	2.501.605	Fengycin	40	BGC0001095
Region 1.7	RIPP-like	2,554,758	2,564,481	–	–	
Region 1.8	RIPP-like	2,668,030	2,678,290	–	–	
Region 1.9	NRPS	2,695,344	2,742,272	–	–	
Region 1.10	CDPS	3,123,159	3,143,878	–	–	
Region 1.11	NRPS	3,305,801	3,365,759	Polyoxypeptin	5	BGC0001036
Region 1.12	terpene	3,496,213	3,518,066	Molybdenum cofactor	17	BGC0000916
Region 2.1	ladderane	1	25,790	S-layer glycan	20	BGC0000794
Region 2.2	ranthipeptide	48,950	70,356	–	–	
Region 3.1	CDPS	132,856	153,575	–	–	
Region 3.2	NRPS	245,202	292,800	–	–	
Region 4.1	RIPP-like	7553	19,769	–	–	
Region 4.2	Lanthipeptide-class-ii	29,816	52,923	–	–	

^a^Identification numbers of the most similar gene clusters from the MIBiG BGC database

The antiSMASH program was used to predict the secondary metabolite biosynthetic gene clusters. Different colored blocks represent genes with different functions; the genes indicated in dark red, light red, blue, green, and gray are core biosynthetic, additional biosynthetic, transport-related, regulatory, and other genes, respectively.

### Antifungal activity of *B. cereus* CF4-51 extracts

**Fig. 3:**
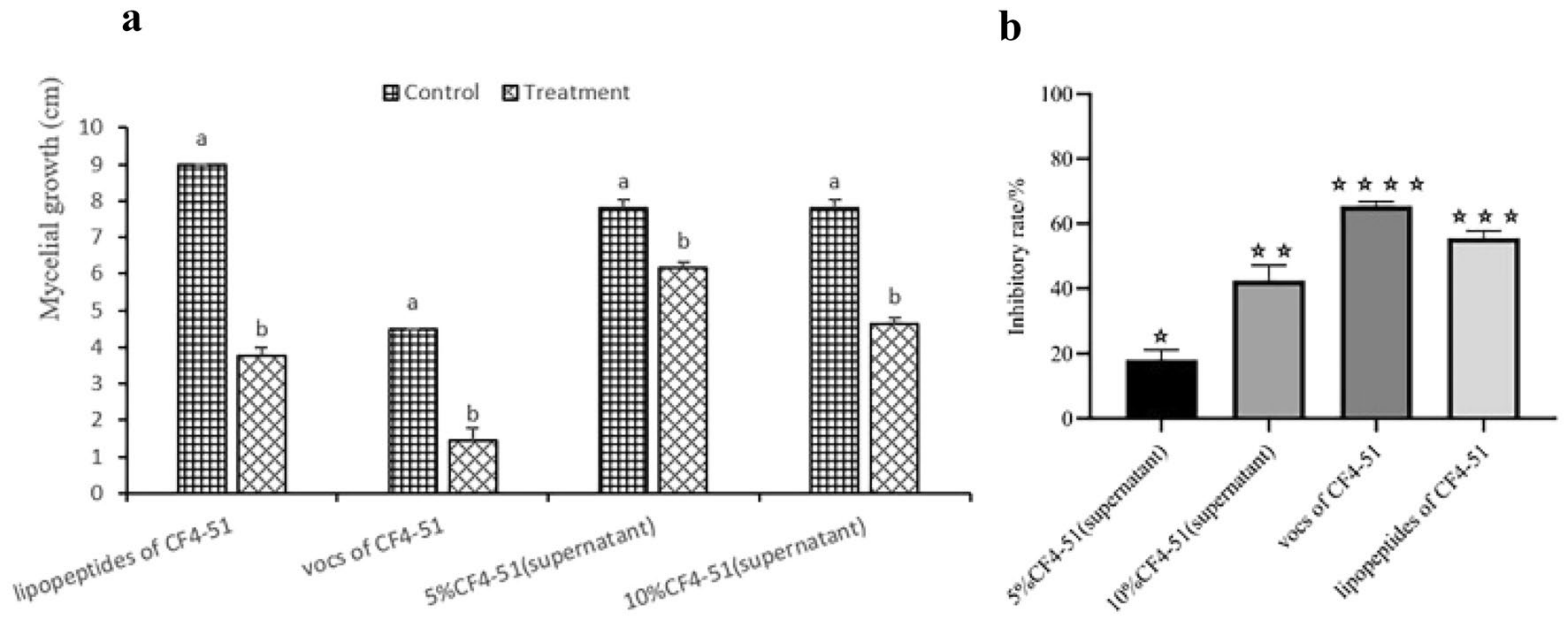
Antifungal activity of Bacillus B. cereus CF4-51 extracts. **a** Mycelial growth of *S. sclerotiorum* after different treatments; **b** inhibitory effects of different treatments on *S. sclerotiorum* mycelial growth

The co-culturing of *B. cereus* and *S. sclerotiorum* revealed the significant inhibitory effects of strain CF4-51 on *S. sclerotiorum* mycelial growth. To identify the *B. cereus* antifungal extracts, *S. sclerotiorum* was treated with bacterial extracts. Previous research confirmed that lipopeptide antibiotics can alter membrane permeability. In the current study, we identified many gene clusters involved in the synthesis of ranthipeptides and lanthipeptides (class II). Subsequently, we extracted the lipopeptides from *B. cereus* CF4-51 as previously described and then analyzed their antagonistic activities. These lipopeptides inhibited *S. sclerotiorum* mycelial growth, with an inhibition zone of 2.5 ± 0.08 cm. Therefore, the extracted *B. cereus* CF4-51 lipopeptides were analyzed by FPLC. The lipopeptides produced by CF4-51 were identified as fengycin on the basis of a comparison with the peak for pure fengycin. The results indicated the strain CF4-51 cell-free supernatants (5% and 10%) and VOCs、lipopeptides inhibited *S. sclerotiorum* mycelial growth by 18.20 ± 0. 02%, 42.40 ± 0.03% and 65.40 ± 0.01%、55.60 ± 0.01%, respectively. Accordingly, the VOCs had a greater inhibitory effect on *S. sclerotiorum* mycelia than other extracts (Fig. 3).

### Identification and antifungal activity of VOCs produced by *B. cereus* CF4-51

The active components of the VOCs were determined by SPME and GC–MS/MS analyses. The same volatiles in the LB medium and substances with relative contents less than 0.5% were filtered out. This revealed very clear separation between the control and strain CF4-51. Forty-five CF4-51 VOC components were identified, including 4 esters, 3 ketones, 3 ethers, 11 alkanes, 3 naphthalenes, 3 alcohols, 4 amines, 2 phenols, 2 benzenes, 1 organic acid, 1 aldehyde, and 1 furanone (Supplementary Table S2).

The mycelial inhibition assays results of five chemicals showed that after 3 days, all of the five chemicals fully inhibited mycelial extension of *S. sclerotiorum* in vitro at concentration of 1000μL/L. In addition, 100 µL/L 1,2-Benzenedicarboxylic acid, bis(2-methylpropyl) ester also fully inhibited the mycelium extension. The inhibition rate of the other four chemicals was lower than 1,2-Benzenedicarboxylic acid, bis(2-methylpropyl) ester. This results indicated that these five VOCs play important role in inhibiting mycelium extension, the 1,2-Benzenedicarboxylic acid, bis(2-methylpropyl) ester was the most potential component (Table 2).

**Table 2:** Inhibition effects of selected VOCs on *S. sclerotiorum*

Volatile compound	Mycelial growth (cm) at different concentrations of volatiles				Inhibition percentages at different concentrations of volatiles（%）			
	1 µL/L	10 µL/L	100 µL/L	1000 µL/L	1 µL/L	10 µL/L	100 µL/L	1000 µL/L
Cyclododecane	4.50±0.00a	2.98±0.32b	1.22±0.16bc	0.00±0.00a	0.00±0.00a	0.34±0.07bc	0.73±0.04b	100±0.00a
Dibutyl phthalate	4.50±0.00a	2.80±0.18bc	1.13±0.03bc	0.00±0.00a	0.00±0.00a	0.37±0.04bc	0.75±0.01b	100±0.00a
1,2-Benzenedicarboxylic acid, bis(2-methylpropyl) ester	4.50±0.00a	1.50±0.05d	0.00±0.00d	0.00±0.00a	0.00±0.00a	0.67±0.01a	100±0.00a	100±0.00a
Heptadecane	4.50±0.00a	2.50±0.43c	0.78±0.46c	0.00±0.00a	0.00±0.00a	0.44±0.09b	0.83±0.10b	100±0.00a
2-Pentadecanone,6,10,14-trimethyl-	4.50±0.00a	3.18±0.20b	1.30±1.30b	0.00±0.00a	0.00±0.00a	0.29±0.04c	0.71±0.05b	100±0.00a
Control (sterile distilled water)	4.50±0.00a	4.50±0.00a	4.50±0.00a	4.50±0.00a				

Different letters (a, b,c) indicated the significant differences (*p* value < 0.05)

### Strain CF4-51 VOCs disrupt the *S. sclerotiorum* cell membrane

**Fig. 4:**
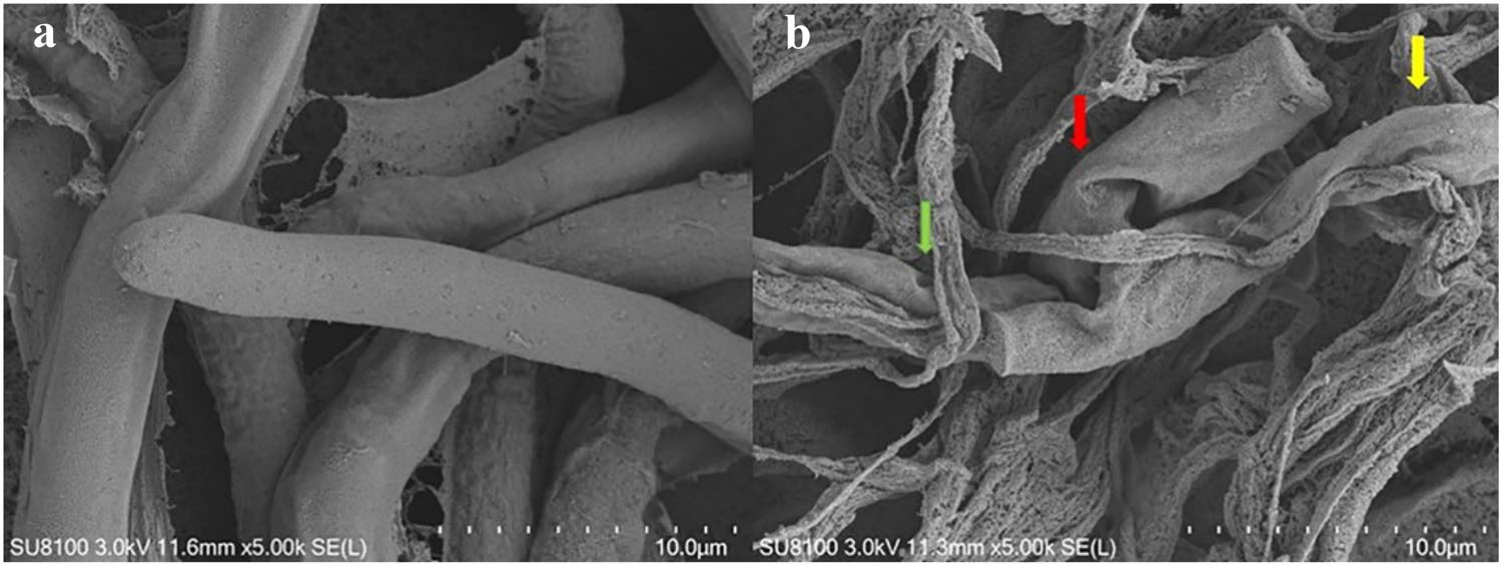
Scanning electron micrographs of *Sclerotinia sclerotiorum* co-cultured with CF4-51 volatile organic compounds (VOCs). **a** Untreated control mycelia. **b** Mycelia treated with VOCs:red arrows indicate empty segments; yellow arrows indicate a flaccid hyphae; green arrows indicate a retracted protoplasm

To clarify how the VOCs affect mycelial growth, the *S. sclerotiorum* mycelia were examined by scanning electron microscopy. Regular length and intact cell walls with uniform composition and structure were present in the hyphae of *S. sclerotiorum* in the control group (Fig. 4a), In contrast, A 5-day treatment of the *S. sclerotiorum* mycelia with the strain CF4-51 VOCs exhibited substantial structural destruction (Fig. 4b). In detail, some of the hyphae became expanded, and the formation of empty segments was presented (red arrows, Fig. 4b). Treated mycelium appeared with a more flaccid hyphae, and the surface of the cell walls became uneven (yellow arrows, Fig. 4b). In addition, thin or gapped structures presenting a retracted protoplasm were seen in Fig. 4b (green arrows). The broken structures might lead to the leakage of cytoplasmic components. These results indicate that the CF4-51 VOCs damaged the *S. sclerotiorum* mycelial cell wall structure. We speculated that the VOCs also altered the cell membrane permeability, leading to the external flow of cytoplasm and the collapse of the cell wall.

### Gene expression analysis by qRT-PCR

**Fig. 5:**
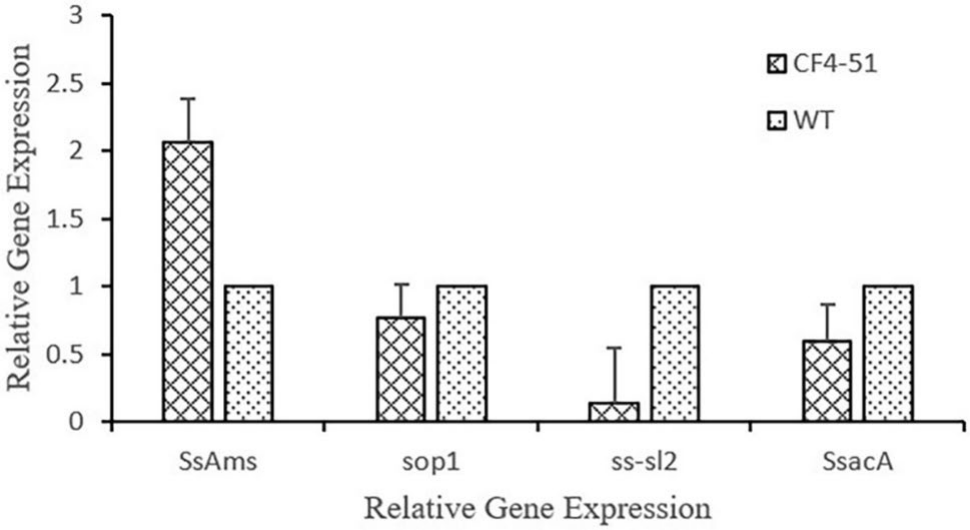
Effects of *Bacillus cereus* CF4-51 VOCs on the expression of genes related to the formation of sclerotia

Previous study demonstrated that genes *Ss-sl2* (Yu et al. 2012), *SOP1* (CuiCui 2013), *SsAMS2* (Liu et al. 2018), *SsSac1* (Wayne and Rollins 2007) contributed to cell wall structure or involved in the formation of sclerotia. To elucidate the molecular mechanism underlying the inhibitory effects of the VOCs on the formation of sclerotia, we analyzed the expression levels of these genes. In the current study, the qRT-PCR data indicated the exposure to *B. cereus* CF4-51 VOCs significantly upregulated *SsAMS* expression, *SsAMS* was up-expressed with 2.05-folds increase compared to control, whereas it had the opposite effect on the expression of the genes encoding *SOP1*, *SsSacA*, and *Ss-sl2*. *SOP1*, *SsSacA*, and *Ss-sl2* were down-regulated with 0.61-, 1.84- and 0.25-folds decrease, separately. These results suggest that strain CF4-51 VOCs inhibit sclerotial formation by altering the expression of four genes (Fig. 5).

## Discussion

In this work, we isolated *B. cereus* CF4-51 as a biocontrol strain potentially useful for controlling sclerotinia rot of sunflower. The VOCs and lipopeptides extracted from *B. cereus* CF4-51 can inhibit *S. sclerotiorum* mycelial growth by regulating the expression of growth-related genes. Genome analysis indicated there are many gene clusters related to the synthesis of secondary metabolites in the genome of *B. cereus* CF4-51. In addition, SPME and GC–MS/MS analyses showed that many VOCs are present in *B. cereus* CF4-51.We found that the inhibition of *S. sclerotiorum* mycelial growth by *B. cereus* CF4-51 is associated with the VOCs produced by the bacterial strain.

Previous studies have demonstrated that VOCs typically comprise complex mixtures of low-molecular-weight compounds that can be used to protect crops against plant diseases (Hung et al. 2015). These metabolites are much more environmentally friendly and innocuous than synthetic chemical pesticides, because they are rapidly biodegraded (Schalchli et al. 2016). For example, the benzaldehyde (Tahir et al. 2017), 2-undecanone, dodecane could inhibit the mycelial extension of many fungal plant pathogens, such as *Alternaria alternata* (Groenhagen et al. 2013), *Achromobacter* sp., and *F. oxysporum* (Minerdi et al. 2009) etc. According to the above report, compounds identified from CF4-51, such as 2-pentadecanone, Cyclododecane, and Benzaldehyde,2-nitro-,Diaminomethylidenhydrazone were potential in inhibiting *Fusarium oxysporum* and *Ralstonia solanacearum*. Among 54 identified chemicals with strong antifungal effects, the antifungal efficiency of 2-Pentadecanone,6,10,14-trimethyl-,1,2-Benzenedicarboxylic acid,bis(2-methylpropyl) ester, Dibutyl phthalate, Cyclododecane, Heptadecane have been verified. These results indicated that VOCs from biocontrol bacteria were potential in plant disease management.

There is considerable interest in the utility of VOCs produced by microorganisms for controlling plant diseases (Schalchli et al. 2016; Rajani et al. 2020). Volatile organic compounds (VOCs) are usually lipophilic substances that are released through biofilms and into the atmosphere or soil. The fungal pathogens were inhibited by the bio-fumigation of VOCs (Pichersky et al. 2006). We also confirmed that VOCs of *B. cereus* CF4-5 inhibited many soil-born fungal pathogens. Some VOCs also serve as signal substances for communication between organisms and within organisms as well as between cells of the same organism (Kai et al. 2009). Therefore, VOCs of biocontrol bacteria manage plant by multiple mechanism and should be used in enclosed environment, such as greenhouse and warehouse.

Fungal cell membrane are critical for cell integrity and normal physiological activities (Malinsky and Opekarová 2016). Breakage of cell membrane can result in the leakage of cytoplasmic compounds, which can alter the shape and structure of hyphae (Boukaew and Prasertsan 2014). The majority of studies focus on mycelia morphology and penetration and spore germination at the cell level (Qili et al. 2010; Kong et al. 2014; Wu et al. 2015; Gotor-Vila et al. 2017; Zhenfeng et al. 2017). The VOCs of many biocontrol bacteria can modulate the structural features of plant pathogenic fungi (Minerdi et al. 2009; Ando et al. 2010; Glare et al. 2012; Viviane et al. 2020). In this study, VOCs altered the hyphae internal structures and surface morphology in the majority of *S. sclerotiorum* cells. Meanwhile, *S. sclerotiorum* cells exposed to VOCs formed swollen part of hyphae with defective ability, leading to aborted invasion to the plant barrier. Moreover, the VOCs affected the expression of many genes (e.g., *SsSac*, *Ss-Sl2*, *SsSOP1*, and *SsAMS2*) associated with the polar growth and integrity of fungal hyphae and the formation of sclerotia (Takayama and Takahashi 2007; Takayama et al. 2010; Trickey et al. 2013; Liu et al. 2018). These results indicate that the reason why VOCs inhibit the mycelium extension is that VOCs impact the expression of genes involved in integrity and polar growth of hyphae.

Bacteria genomes can be divided into three categories according to genome size, including small genome (0.5–2 Mb), medium genome (2–5 Mb), and large genome (5–10 Mb) (Ochman and Davalos 2006). Bacteria with large genome usually produce more metabolites and can tolerate more stress (Ranea et al. 2004) and utilize various carbon source than bacteria with small genomes (Juan 2012). These features were favorable for the stable colonization and production of antagonistic compounds by bacteria (Linchong et al. 2012; Cheng et al. 2014; Wei 2014; Zhuanzhuan et al. 2015; Zongsuo et al. 2017). The strain CF4-51 genome comprises 5.35 Mb (i.e., a large genome). In addition, it contains 230 genes encoding enzymes related to carbohydrate synthesis and 315 genes involved in carbohydrate transport and metabolism. Thus, strain CF4-51 may be able to use diverse carbon sources, enabling rapid growth and reproduction. We also discovered 18 gene clusters, which included 708 genes contributing to the synthesis of secondary metabolites. Therefore, CF4-51 may produce antagonistic compounds and survive under adverse environmental conditions Accordingly, CF4-51 is a promising bacterial strain for controlling sclerotinia rot.

Lipopeptides produced by multiple *Bacillus* spp., including surfactin, iturin, and fengycin, are important antagonistic compounds which control plant disease by antagonism, inducing resistance and growth promotion (Menkhaus et al. 1993; Huijun et al. 2012). Previous studies have demonstrated that NRPSs, which consist of five subunits encoded by *ppsA*, *ppsB*, *ppsC*, *ppsD*, and *ppsE*, can catalyze the production of lipopeptides from amino acids (Nakano et al. 1988; Ruckert et al. 2011). Our analysis of the *B. cereus* CF4-51 genome revealed the presence of the depsipeptide gene clusters *ppsA*, *ppsB*, *ppsC*, *ppsD*, and *ppsE* related to fengycin synthesis (Marahiel 1997; Peypoux et al. 1999). We also identified fengycin among the *B. cereus* CF4-51 metabolites during our FPLC analysis. These findings imply that *B. cereus* CF4-51 synthesizes fengycin, which is important for inhibiting *S. sclerotiorum* hyphal growth and the production of sclerotia.

In conclusion, our study not only highlights the important role of *Bacillus cereus* CF4-51 in antagonizing and inhibiting the growth of *Sclerotinia sclerotiorum*, but also suggests that VOCs generated by *Bacillus cereus* play an indispensable role in the inhibition of pathogen.

## Supplementary Information

Below is the link to the electronic supplementary material.

**Table S1**: Details regarding the qRT-PCR primers. **Table S2**: Volatile organic compounds produced by Bacillus cereus CF4-51. (DOCX 33 KB)

## Data Availability

The authors confirm that the data supporting the findings of this study are available within the article and its supplementary materials.
