# Supplementary material for: Genomic and metabolic features of Bacillus cereus, inhibiting the growth of Sclerotinia sclerotiorum by synthesizing secondary metabolites
Source: Arch Microbiol. 2022 Dec 1;205(1):8. doi: 10.1007/s00203-022-03351-5 (PMC9715469; doi:10.1007/s00203-022-03351-5)
Supplement: Supplementary file 1 — Table S1: Details regarding the qRT-PCR primers. Table S2: Volatile organic compounds produced by Bacillus cereus CF4-51. (DOCX 33 KB) [file 203_2022_3351_MOESM1_ESM.docx]

**Supplementary Table 1** Details regarding the qRT-PCR primers

| Gene Name | Primer name Sequence | Primer name Sequence |
| --- | --- | --- |
|  | ss-sl2F | 5'-GAA ACACCGTGACAGCGAG-3' |
| ss-sl2 | ss-sl2R | 5'-GCCCATTTCCAGCAGGT-3' |
|  | SsSac-F | 5'-GTTGGAGCATGCCGAACTAT-3 |
| SsSac | SsSac-R | 5'-CGATCAAAGCAGTCATGTTGC-3 |
|  | SsAms-F | 5'-CCACCTCGTATCTCCTCAT-3 |
| SsAms | SsAms-R | 5'-GGCATAGTAGTAGCAACCTTA-3 |
|  | Sop1-F | 5'-TTCCAAGTGTTGTTCCTAATGC-3' |
| Sop1 | Sop1-R | 5'GAGAGTGATGAATGCGGTAATAAC-3' |
|  | tubulin-F | 5'-TTGGATTTGCTCCTTTGACCAG-3 |
| tubulin | tubulin-R | 5'-AGCGGCCATCATGTTCTTAGG-3 |

**Supplementary Table 2** Volatile organic compounds produced by *Bacillus cereus* CF4-51

| Chemical class | Retention Time(min) | Area Pct | Library/ID | Chemical formula | CAS | Qual | Reference |
| --- | --- | --- | --- | --- | --- | --- | --- |
| Esters | 24.0751 | 0.5016 | Decanoic acid, 2,8-dimethyl-, methyl ester | C13H26O2 | 055030-52-9 | 91 |  |
|  | 24.7032 | 0.5465 | Cyclopropane, octyl- | C11H22 | 001472-09-9 | 62 |  |
|  | 26.0177 | 0.6243 | Ethyl 13-methyl-tetradecanoate | C16H32O2 | 1000336-61-5 | 93 |  |
|  | 33.9098 | 1.9953 | 1,2-Benzenedicarboxylic acid, bis(2-methylpropyl) ester | C16H22O4 | 000084-69-5 | 80 |  |
| Ketones | 24.4403 | 0.5233 | 2-Pentadecanone | C15H30O | 002345-28-0 | 95 | Groenhagen et al., 2013 |
|  | 26.5241 | 0.6632 | 2-Pentadecanone,6,10,14-trimethyl- | C18H36O | 000502-69-2 | 98 |  |
|  | 28.4034 | 0.8582 | 1H-Indene-1,3(2H)-dione, 2-hydroxy-2-(9-methoxy-9H-fluoren-9-yl)- | C23H16O4 | 050616-96-1 | 96 |  |
| Ethers | 32.8679 | 1.5993 | 15-Crown-5 | C10H20O5 | 033100-27-5 | 43 |  |
|  | 33.2136 | 1.8713 | 12-Crown-4 | C8H16O4 | 000294-93-9 | 38 |  |
|  | 29.2115 | 1.0055 | Propane, 1,1'-oxybis[2,3-dichloro- | C6H10OCl4 | 007774-68-7 | 41 |  |
|  | 34.9663 | 4.3517 | Ethanol, 2-(vinyloxy)- | C4H8O2 | 000764-48-7 | 27 |  |
| Alkane | 24.2601 | 0.5093 | Trimethyl-2-thienylsilane | C7H12SSi | 018245-28-8 | 83 |  |
|  | 25.0781 | 0.5524 | Tridecanenitrile | C13H25N | 000629-60-7 | 35 |  |
|  | 25.1755 | 0.5554 | Hexasiloxane, 1,1,3,3,5,5,7,7,9,9,11,11-dodecamethyl- | C12H36O5Si6 | 000995-82-4 | 43 |  |
|  | 25.8473 | 0.5984 | Cyclohexasiloxane, dodecamethyl- | C12H36O6SI6 | 000540-97-6 | 45 |  |
|  | 27.269 | 0.7453 | Cyclododecane | C12H24 | 000294-62-2 | 97 | Minerdi et al., 2010 |
|  | 27.4491 | 0.7792 | Heptasiloxane,1,1,3,3,5,5,7,7,9,9,11,11,13,13-tetradecamethyl- | C14H44O6Si7 | 019095-23-9 | 38 |  |
|  | 27.5611 | 0.8209 | Benzene,1,1'-methylenebis[4-methyl- | C15H16 | 004957-14-6 | 83 |  |
|  | 29.8542 | 1.0754 | (4-Acetylphenyl)phenylmethane | C15H14O | 000782-92-3 | 43 |  |
|  | 32.3226 | 1.4461 | 1-Propanamine, N1-methyl-2-methoxy | C4H11NO | 1000198-01-0 | 35 |  |
|  | 31.2126 | 1.2659 | Cyclotetradecane | C14H28 | 000295-17-0 | 95 |  |
|  | 34.6985 | 4.1003 | Octasiloxane,1,1,3,3,5,5,7,7,9,9,11,11,13,13,15,15-hexadecamethyl- | C16H50O7Si8 | 019095-24-0 | 45 |  |
| naphthalene | 25.7354 | 0.5827 | Naphthalene, 1,4,6-trimethyl- | C13H14 | 002131-42-2 | 95 |  |
|  | 26.4218 | 0.6553 | Naphthalene, 1,6,7-trimethyl- | C13H14 | 002245-38-7 | 74 |  |
|  | 27.1911 | 0.7098 | Naphthalene, 2,3,6-trimethyl- | C13H14 | 000829-26-5 | 95 |  |
| Alcohols | 24.5425 | 0.5285 | 3-Octadecene, (E)- | C18H36 | 007206-19-1 | 83 |  |
|  | 25.4481 | 0.5657 | n-Tridecan-1-ol | C13H28O | 000112-70-9 | 91 |  |
|  | 26.7724 | 0.6738 | Butanedial, dioxime | C4H8N2O2 | 002580-71-4 | 25 |  |
| amine | 27.6877 | 0.8341 | 3-Hydroxy-N, N-dimethylpropanamide | C5H11NO2 | 029164-29-2 | 38 |  |
|  | 28.9292 | 0.8977 | Dimethylamine | C2H7N | 000124-40-3 | 35 |  |
|  | 34.0656 | 2.3529 | Acetamide, N-(2-phenylethyl)- | C10H13NO | 000877-95-2 | 49 |  |
|  | 34.2603 | 3.301 | 2,2-Dimethyl-N-phenethylpropionamide | C13H19NO | 062056-54-6 | 49 |  |
| Phenols | 26.1735 | 0.6339 | Phenol, 2-(1-methylethyl)- | C9H12O | 000088-69-7 | 93 |  |
|  | 30.5699 | 1.1481 | Phenol, 2,4-bis(1,1-dimethylethyl)- | C14H22O | 000096-76-4 | 97 |  |
| benzene | 28.0041 | 0.8355 | 3,3'-Dimethylbiphenyl | C14H14 | 000612-75-9 | 87 |  |
|  | 25.5455 | 0.5687 | 1,1'-Biphenyl, 3-methyl- | C13H12 | 000643-93-6 | 60 |  |
| Organic acids | 24.635 | 0.5342 | Adipic dihydroxamic acid monohydrate | C6H12N2O4 | 004726-83-4 | 30 |  |
| Aldehydes | 24.9515 | 0.5488 | Benzaldehyde, 2-nitro-, diaminomethylidenhydrazone | C8H9N5O2 | 102632-31-5 | 41 | Tahiret al.,2017 |
| Furanone | 28.6857 | 0.8972 | Dibenzofuran | C12H8O | 000132-64-9 | 46 |  |
| other | 24.1482 | 0.5071 | 1,10-Undecadiene | C11H20 | 013688-67-0 | 83 |  |
|  | 35.8085 | 5.403 | Dibutyl phthalate | C16H22O4 | 000084-74-2 | 93 |  |
|  | 29.4306 | 1.0202 | Tetradecanenitrile | C14H27N | 000629-63-0 | 50 |  |
|  | 30.901 | 1.1617 | Fluorene | C13H10 | 000086-73-7 | 62 |  |
|  | 32.605 | 1.5077 | Indole | C8H7N | 000120-72-9 | 94 |  |
|  | 32.7608 | 1.5195 | Phenethylamine,3,4,5trimethoxy-.alpha.-methyl- | C12H19NO3 | 001082-88-8 | 35 |  |

B

A

B

A

B

A

B

A
